# The CTLA-4 Pathway in Human Disease: Molecular Mechanisms and Therapeutic Targeting

**DOI:** 10.3390/genes17050574

**Published:** 2026-05-18

**Authors:** Simone Negrini, Stefania Nicola, Iuliana Badiu, Anna Quinternetto, Ilaria Vitali, Luca Lo Sardo, Luisa Brussino

**Affiliations:** 1 Department of Medical Sciences, University of Turin, 10124 Turin, Italy; stefania.nicola@unito.it (S.N.); iuliana.badiu@gmail.com (I.B.); annaquinternetto@gmail.com (A.Q.); ilariavitali1@gmail.com (I.V.); lucalosardoallergologo@gmail.com (L.L.S.); luisa.brussino@unito.it (L.B.); 2 Immunology and Allergy Unit, AO Ordine Mauriziano di Torino, 10128 Turin, Italy

**Keywords:** CTLA-4, immune regulation, regulatory T cells, CTLA-4 insufficiency, LRBA deficiency, trans-endocytosis, checkpoint inhibition, immunotherapy

## Abstract

Background/Objectives: CTLA-4 is a key checkpoint of peripheral immune regulation, yet its biology cannot be reduced to inhibitory signaling alone. This review discusses CTLA-4 as a dynamic regulatory pathway shaped by ligand handling, intracellular trafficking, recycling, and cell-type-specific function, and examines how these features link molecular mechanism to human disease and therapy. Methods: We synthesized the structural, mechanistic, translational, and clinical literature spanning CTLA-4 molecular biology, cell-type-specific function, inborn errors of immunity, polygenic autoimmunity, transplantation, cancer immunotherapy, and immune-related adverse events. Results: CTLA-4 function depends on surface availability, trans-endocytosis of CD80/CD86, and tight control of endosomal trafficking. These features help explain why CTLA-4 haploinsufficiency, LRBA deficiency, and DEF6 deficiency converge clinically despite different upstream lesions, and why subtler CTLA-4 variation contributes to polygenic autoimmunity. Therapeutic studies also provide mechanistic insight. Abatacept can partly replace pathway function in monogenic disease, whereas belatacept highlights the limits of ligand blockade when endogenous coinhibition is also lost. In oncology, anti-CTLA-4 antibodies act through a more complex interplay involving checkpoint blockade, Fc biology, intratumoral Treg depletion, and receptor recycling. Emerging next-generation agents aim to retain antitumor activity while reducing systemic toxicity through more selective use of these mechanisms. Conclusions: Rather than a static inhibitory receptor, CTLA-4 is better viewed as a context-dependent regulatory pathway whose function depends on trafficking, surface availability, and cellular context. This perspective links molecular mechanism to clinical phenotype and supports more precise CTLA-4-targeted therapy.

## 1. Introduction

CTLA-4 has become one of the most informative pathways in human immunology because it lies at the crossroads of tolerance, inflammation, and therapy. First described as a negative regulator of T-cell activation, it is now recognized as a central checkpoint of peripheral immune homeostasis whose disruption can surface in very different clinical settings. Germline defects in CTLA-4 itself, or in genes that control its trafficking such as *LRBA* and *DEF6*, lead to syndromes of immune dysregulation marked by lymphoproliferation, autoimmunity, humoral defects, and organ-specific inflammation. At the other end of the spectrum, deliberate pharmacologic interference with the same pathway has reshaped cancer therapy while also provoking immune-related toxicities that echo important features of inherited CTLA-4 insufficiency. Transplantation and common polygenic autoimmunity occupy the space between these extremes. Across these settings, CTLA-4 provides a useful framework for understanding human immune regulation across genetics, mechanism, and therapy.

However, describing CTLA-4 simply as an “inhibitory receptor” is no longer sufficient. The shorthand is useful, but incomplete, because CTLA-4 biology depends heavily on intracellular trafficking, dynamic surface mobilization, ligand handling, and the cell in which the pathway is deployed. Most CTLA-4 resides inside the cell and must be mobilized to the surface in a tightly regulated way. Its function is therefore shaped not only by expression level, but also by endocytosis, recycling, trans-endocytosis of CD80/CD86, and the molecular machinery that preserves or depletes the usable surface pool. This helps explain why defects in gene dosage, ligand binding, endosomal recycling, or antibody-induced lysosomal degradation can converge on overlapping clinical phenotypes. It also helps explain why the pathway behaves differently across immune contexts. In regulatory T (Treg) cells and follicular regulatory T (Tfr) cells, CTLA-4 has a dominant cell-extrinsic role in restraining costimulation; in conventional T cells and CD8 T cells, its contribution is more context-dependent and more tightly linked to activation state, differentiation, and tissue environment. CTLA-4 is better viewed as a dynamic regulatory system rather than as a receptor with a single fixed function.

We begin with the core molecular biology of the CTLA-4 pathway, including structure, ligand binding, intracellular distribution, recycling, and trans-endocytosis. We then examine how these mechanisms play out across specific cell types and disease settings, from monogenic immune dysregulation to autoimmunity, transplantation, cancer immunotherapy, and immune-related adverse events. Throughout the review, surface availability serves as a practical guiding concept because it links molecular mechanism to clinical phenotype.

**Figure 1:**
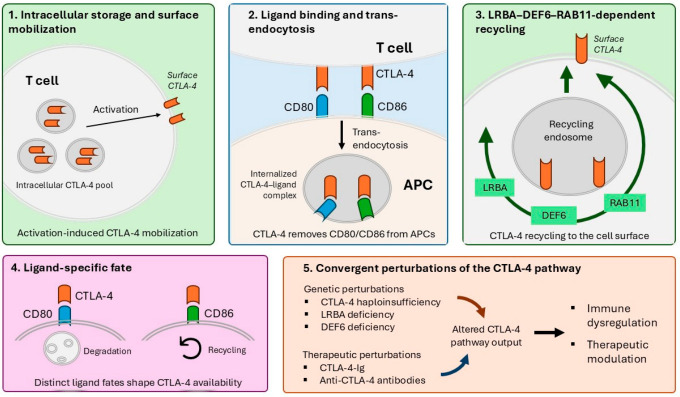
CTLA-4 as a dynamic regulatory pathway: trafficking, ligand handling, recycling, and convergent disease and therapeutic perturbations.

The central mechanistic framework that links CTLA-4 mobilization, ligand capture, recycling, disease-associated disruption, and therapeutic modulation is summarized schematically in Figure 1.

CTLA-4 is predominantly stored in intracellular vesicles and is rapidly mobilized to the T-cell surface following activation. At the immune synapse, CTLA-4 binds CD80 and CD86 and removes these ligands from antigen-presenting cells through trans-endocytosis, thereby limiting CD28-mediated costimulation. After ligand capture, CTLA-4 trafficking is regulated by endosomal sorting and recycling pathways involving LRBA, DEF6, and RAB11. Experimental evidence supports distinct ligand-associated fates, with CD80 capture being more closely linked to CTLA-4 degradation, whereas CD86 capture is more compatible with receptor recycling. Monogenic disorders such as CTLA-4 haploinsufficiency, LRBA deficiency, and DEF6 deficiency converge on reduced functional CTLA-4 surface availability. Therapeutic interventions modulate the same pathway through extracellular CD80/CD86 sequestration (CTLA-4-Ig) and checkpoint blockade, including Fc-dependent effects and altered intratumoral Treg activity during anti-CTLA-4 therapy.

## 2. Core Molecular Biology of the CTLA-4 Pathway

### 2.1. Receptor Structure, Dimerization, and Ligand Binding

CTLA-4 is a member of the immunoglobulin superfamily and functions as a pre-existing covalent homodimer rather than a receptor that assembles only upon ligand engagement [1,2,3,4,5,6]. Its extracellular domain adopts a V-set immunoglobulin fold, and each monomer contributes a ligand-binding surface centered on the canonical MYPPPY motif, allowing the receptor dimer to engage B7 ligands with high avidity [1,2,3,4,5,6]. Early biochemical work established that CTLA-4 is linked by an intermolecular disulfide bond in the membrane-proximal stalk and that the recombinant dimeric CTLA-4 ectodomain used in those studies forms a 2:2 complex with CD86, thereby demonstrating receptor bivalency; importantly, this construct should not be confused with the distinct soluble splice isoform discussed in Section 2.8 [3].

The structural basis of ligand recognition was defined by the crystal structures of the human CTLA-4/B7-1 (CD80) and CTLA-4/B7-2 (CD86) complexes [1,2]. In the CTLA-4/B7-1 structure, the total buried surface area is approximately 1255 Å^2^, and the interface shows unusually high shape complementarity (Sc 0.74–0.77), despite being relatively small by general protein–protein interaction standards [1]. The FG loop containing the MYPPPY sequence contributes roughly 400 Å^2^ of the CTLA-4 interface, underscoring the central role of this motif in ligand binding [1].

The binding asymmetry between CD80 and CD86 has important functional consequences. Biophysical analyses showed that B7-1 binds CTLA-4 more strongly than B7-2, whereas CD28 engages both ligands much more weakly and, unlike CTLA-4, behaves as a functionally monovalent homodimer [4,5]. In classical surface plasmon resonance analyses, monomeric CTLA-4 bound CD80 with a dissociation constant in the submicromolar range (about 0.2–0.4 μM), whereas the corresponding interaction between CD28 and CD80 was markedly weaker, in the low- to mid-micromolar range depending on assay format [4,5]. Thus, CTLA-4 is structurally optimized not only for ligand binding, but for high-avidity competition against CD28, especially in the case of CD80 [4,5]. This distinction becomes increasingly important in later sections, where ligand-specific intracellular fate and recycling efficiency emerge as major determinants of function.

Structural work also supports the view that CTLA-4 presents a largely pre-formed B7-binding surface. High-resolution analysis of the unliganded human homodimer showed that ligand-bound and unbound CTLA-4 are remarkably similar, consistent with a predominantly rigid-body mode of recognition, particularly for B7-1 [6]. This strengthens the idea that the major functional determinants of the pathway lie not in ligand-induced receptor remodeling, but in ligand availability, receptor valency, and post-binding trafficking.

The same front-sheet surface used for B7 recognition is also targeted by ipilimumab, whose crystal structure with CTLA-4 shows direct steric overlap with the B7-binding face, including the MYPPPY-containing region [7]. Patient-associated variants affecting this surface can therefore disrupt both physiological ligand recognition and the molecular logic of checkpoint regulation. A clinically instructive example is the recurrent p.Tyr139Cys mutation, which maps to the MYPPPY region and critically impairs CD80/CD86 binding with loss of suppressive function [8]. Structural studies therefore support a model in which CTLA-4 is a rigid, pre-formed, bivalent receptor whose geometry and avidity are central to ligand competition and endocytic function [1,2,3,4,5,6,7,8].

### 2.2. Cellular Expression and Intracellular Distribution

CTLA-4 expression is highly cell-type- and activation-dependent. The clearest and most consistent distinction is between the regulatory compartment, where CTLA-4 is constitutively associated with cellular identity and homeostasis, and conventional T cells, where it is predominantly an activation-induced molecule [9,10,11,12]. This expression pattern is fundamental to understanding why CTLA-4 contributes both to steady-state immune restraint and to inducible negative feedback after T-cell activation.

Among CD4+ T cells, FOXP3+ regulatory T cells (Treg) express CTLA-4 constitutively and at higher levels than resting conventional T cells [9,10]. In mice, CTLA-4 upregulation is tightly linked to the rapid homeostatic turnover of Foxp3+ Treg cells, supporting the view that CTLA-4 is not merely a marker of this compartment but an indicator of ongoing physiological activation [9]. Human studies point in the same direction: within peripheral blood CD4+CD25+ cells, the CTLA-4-positive fraction is enriched for suppressive activity and for higher FOXP3 expression, indicating that surface-accessible CTLA-4 identifies a more functionally homogeneous regulatory subset [10].

By contrast, conventional CD4+ T cells acquire CTLA-4 mainly after stimulation. Activation of human CD4+CD25− cells generates a CTLA-4-high population that initially overlaps strongly with induced FOXP3 expression, although CTLA-4 can persist even when FOXP3 later declines [11]. This shows that CTLA-4 expression is not restricted to natural Treg cells and can emerge de novo in activated conventional T cells under appropriate stimulation conditions [11]. Expression is also quantitatively uneven across lineages: in normal human T cells, CTLA-4 is expressed significantly more strongly in CD4+ than CD8+ T cells, at both transcript and protein levels, in association with greater NFAT1-dependent promoter activity in CD4+ cells [12].

A second defining feature of CTLA-4 is its predominantly intracellular distribution. Only a limited fraction of total CTLA-4 is detectable at the cell surface at any given time, whereas a much larger pool resides in intracellular vesicular compartments [10,11,13]. In human cells, experimental mobilization is often required to reveal substantial surface CTLA-4, indicating that low steady-state surface abundance reflects trafficking behavior rather than absent protein production [10]. This predominantly intracellular localization has important functional consequences: CTLA-4 function depends not simply on whether the receptor is expressed, but on whether it can be delivered to, retrieved from, and returned to the plasma membrane.

### 2.3. Endocytosis, Intracellular Trafficking, and Control of Surface Expression

A defining feature of CTLA-4 biology is that its inhibitory function is regulated not simply by transcription or total protein abundance, but by continuous intracellular trafficking. In contrast to receptors that accumulate stably at the plasma membrane, CTLA-4 is predominantly intracellular and undergoes rapid, constitutive cycling between intracellular compartments and the cell surface [13,14,15,16,17,18]. Early work established that only a small fraction of total CTLA-4 is present at the membrane at any given time and that the receptor is rapidly internalized after surface delivery, helping explain why surface expression remains low despite ongoing biosynthesis [13,16].

The core determinant of this behavior is the tyrosine-based YVKM motif in the cytoplasmic tail. Two early studies showed that CTLA-4 binds the medium chain of the clathrin adaptor complex AP-2 through this motif, whereas CD28 does not [14,15]. In both systems, mutation of the key tyrosine residue markedly reduced adaptor binding, indicating that the YVKM sequence is a direct endocytic sorting signal [14,15]. AP-2 binding requires the unphosphorylated form of this tyrosine, whereas phosphorylation redirects the same region toward SH2-domain-containing signaling proteins such as PI3K and SHP-2/Syp [15]. Thus, the same tail motif acts as a molecular switch between signaling competence and internalization.

Subsequent work clarified that CTLA-4 internalization is an active clathrin- and dynamin-dependent process. Qureshi et al. showed that CTLA-4 is constitutively internalized in a ligand-independent manner, that deletion or mutation of the YVKM-containing tail impairs this process, and that interference with clathrin-mediated endocytosis or dynamin function traps CTLA-4 at the cell surface [16]. Importantly, most surface CTLA-4 internalized within minutes, emphasizing how short-lived the surface pool is [16]. T-cell activation increased CTLA-4 mobilization to the plasma membrane, but did not stabilize it there: the receptor continued to undergo endocytosis even under activated conditions [16].

Once internalized, CTLA-4 enters a branching itinerary involving both recycling and degradation. Qureshi et al. demonstrated overlap of internalized CTLA-4 with Rab11-positive recycling endosomes and direct return of internalized receptor to the surface [16].

CTLA-4 was also degraded in a lysosome-sensitive manner, supporting a model in which receptor expression is maintained by continuous surface delivery, internalization, recycling, and degradation rather than by long-term membrane residence [16]. AP-1 was later shown to associate with CTLA-4 in post-Golgi compartments, providing a likely route for anterograde traffic from the trans-Golgi network toward endosomal or plasma-membrane destinations [17], while Darlington et al. showed that, after stimulation, CTLA-4 can also partition into lipid rafts and co-cluster with TCR/CD3 and GM1 at the immunological synapse, with this redistribution depending on the cytoplasmic tail [18]. Although this does not define the dominant mechanism of CTLA-4 function, it indicates that the transient surface fraction of the receptor can be selectively organized into specialized membrane domains before re-internalization. What matters biologically is not only how much CTLA-4 a cell expresses, but how efficiently the receptor is trafficked through these compartments and returned to the membrane [13,14,15,16,17,18].

### 2.4. LRBA and DEF6 as Regulators of CTLA-4 Recycling

The importance of intracellular trafficking to CTLA-4 biology is illustrated most clearly by LRBA and DEF6, two molecules whose deficiency does not primarily alter CTLA-4 sequence or transcription, but instead impairs its endosomal recycling and thereby reduces its functional availability at the cell surface [19,20,21,22]. These disorders show that CTLA-4 pathway failure may arise not only from mutations in CTLA-4 itself, but also from defects in the intracellular machinery that determines whether internalized receptor is returned to the plasma membrane or diverted toward degradation.

The first major link between LRBA deficiency and CTLA-4 insufficiency came from the observation that patients with *LRBA* mutations have markedly reduced CTLA-4 protein in both FOXP3+ regulatory T cells and activated conventional T cells, together with immune dysregulation responsive to CTLA-4-Ig therapy [19]. In that study, LRBA colocalized with CTLA-4 in recycling endosomes and the trans-Golgi network, and lysosomal blockade restored CTLA-4 levels in LRBA-deficient cells, indicating that LRBA normally protects CTLA-4 from premature lysosomal degradation [19].

This model was refined mechanistically by Janman et al., who showed that CTLA-4 traffics through Rab5-, Rab7-, and Rab11-positive compartments and that Rab11-dependent recycling is a critical determinant of surface re-expression [20]. In LRBA-deficient cells, CTLA-4 recycling was markedly impaired, degradation was increased, and colocalization with Rab11 was reduced [20]. Importantly, constitutively active Rab11 did not rescue the defect, placing LRBA upstream of Rab11-mediated recycling rather than acting as a parallel trafficking factor [20]. Thus, LRBA is not merely permissive for CTLA-4 expression; it helps preserve internalized receptors from degradative routing and supports their return to the cell surface [19,20].

A closely related mechanism operates in DEF6 deficiency. Serwas et al. identified biallelic *DEF6* mutations in patients with systemic autoimmunity and showed that patient T cells had defective trafficking of CTLA-4 to the cell surface despite preserved intracellular production [21]. Mechanistically, disease-associated *DEF6* variants disrupted RAB11-dependent CTLA-4 shuttling, reducing the number of RAB11+CTLA-4+ vesicles in both patient T cells and DEF6-knockout Jurkat cells [21]. Although LRBA and DEF6 are distinct proteins, both converge on defective RAB11-linked recycling and reduced functional CTLA-4 surface expression [20,21].

These studies show that efficient endosomal recycling is central to maintaining functional CTLA-4 at the cell surface. What matters is not only whether CTLA-4 is produced, but whether it returns to the membrane often enough to maintain inhibitory control [19,20,21,22]. Once CTLA-4 reaches the cell surface, its regulatory effect depends largely on how it engages and removes B7 ligands from opposing cells.

### 2.5. Trans-Endocytosis as the Dominant Cell-Extrinsic Mechanism

Among the proposed mechanisms of CTLA-4 action, trans-endocytosis provides the most direct molecular explanation for its cell-extrinsic inhibitory function. Qureshi et al. showed that CTLA-4 can capture CD80 and CD86 from opposing cells, internalize them into intracellular vesicles, and target the acquired ligands for degradation within the CTLA-4-expressing cell [23]. This process is unidirectional and results in impaired costimulatory capacity of the ligand-donor cell [23]. Thus, rather than acting solely by passive ligand competition, CTLA-4 can actively remove the ligands required for CD28-mediated activation.

This mechanism helps explain why CTLA-4 is especially effective in a regulatory context. Hou et al. showed that although strong CD80/CD86-dependent activation of resting human T cells induced robust CTLA-4 expression, CTLA-4 blockade had little effect in that setting [24]. By contrast, when CTLA-4-positive cells were present as regulators, suppression depended on CTLA-4 and was strongly influenced by the amount of available ligand on the APC [24]. At low APC numbers or low ligand density, CTLA-4-dependent suppression was highly effective; at higher ligand levels, suppression was progressively lost, and the degree of inhibition correlated closely with the amount of CD86 remaining on the APC [24]. These results strongly support a quantitative model in which the efficacy of CTLA-4 is predicted by its ability to deplete costimulatory ligands below the threshold required for productive CD28 signaling [24].

The strongest in vivo support for this framework comes from Ovcinnikovs et al. Using ligand-capture assays in vitro and in vivo, they showed that Treg cells constitutively recruit cycling CTLA-4 to the surface ex vivo, rapidly perform trans-endocytosis after TCR stimulation, and outperform activated conventional T cells in vivo [25]. In that study, trans-endocytosis was preferentially associated with ICOS-positive Treg cells, could be triggered by tissue-derived self-antigen, and primarily targeted migratory dendritic cells reaching lymph nodes from peripheral tissues [25]. These findings place trans-endocytosis within a physiological circuit of self-antigen recognition and steady-state control of APC phenotype.

Trans-endocytosis is unlikely to be the only way in which CTLA-4 depletes costimulatory ligands from APCs. Tekguc et al. reported that tailless CTLA-4, lacking the cytoplasmic region required for classical endocytosis, could still promote depletion of CD80/CD86 through CTLA-4-dependent trogocytosis, accompanied by transfer of additional membrane proteins and lipids from APCs to Treg cells [26]. These data do not invalidate the trans-endocytosis model, but indicate that ligand extraction may proceed through more than one cell-contact-dependent process, particularly in stable Treg–APC conjugates [26]. In this setting, trogocytosis appears to be a related but mechanistically distinct process, because it involves acquisition of membrane fragments and bystander molecules rather than selective internalization of receptor–ligand complexes [23,26]. Whether these mechanisms remain fully separable, or partly overlap during physiological CTLA-4-dependent ligand extraction, remains unresolved [23,26].

The available evidence still supports trans-endocytosis as the main mechanism underlying the cell-extrinsic function of CTLA-4, especially in Treg biology [23,24,25,26]. However, the strongest mechanistic and in vivo support for this model derives from murine studies, whereas the human evidence remains more limited and is based mainly on indirect, in vitro, or ex vivo observations [23,24,25,26].

### 2.6. Ligand-Specific Fates: CD80, CD86, and the Importance of Recycling

Kennedy et al. showed that although CTLA-4 can capture both CD80 and CD86 by trans-endocytosis, these ligands do not impose the same intracellular fate on the receptor [27]. Following CD80 capture, CTLA-4 remains associated with the ligand, becomes ubiquitylated, and is preferentially trafficked toward late endosomes and lysosomes. By contrast, after CD86 capture, CTLA-4 dissociates from its ligand in a pH-dependent manner within endosomal compartments and is then efficiently recycled back to the plasma membrane [27]. Thus, CD80 capture tends to consume CTLA-4, whereas CD86 capture preserves the receptor for repeated rounds of ligand removal [27].

This distinction has major functional implications. Kennedy et al. further showed that disease-associated CTLA-4 variants can selectively impair CD86 trans-endocytosis or the subsequent recycling step, linking defective handling of CD86 to human immune dysregulation [27]. In particular, patient-associated missense mutations at Arg70 (Arg70Gln and Arg70Trp) abolished CD86 binding while preserving CD80 interaction, resulting in complete loss of CD86 trans-endocytosis with retained CD80 trans-endocytosis when expressed from the endogenous CTLA-4 locus in human Treg cells [27]. Moreover, LRBA-deficient cells showed largely preserved CD80 trans-endocytosis but markedly impaired CD86 capture, directly linking the recycling defect described in Section 2.4 to a ligand-specific loss of regulatory function [27]. To date, reported pathogenic variants and trafficking defects have predominantly highlighted impaired CD86 handling or CD86-dependent recycling rather than selective defects in CD80 handling, although this pattern may partly reflect ascertainment bias [27]. These observations suggest that an important regulatory feature of CTLA-4 may lie not simply in its ability to bind B7 ligands with high avidity, but in its capacity to undergo serial reuse after CD86 capture.

This mechanistic model is reinforced by functional data from human Treg biology. Halliday et al. showed that, despite CD86 having approximately tenfold lower affinity for CTLA-4 than CD80, CD86 is the dominant ligand supporting Treg proliferation, survival, and maintenance of phenotype when CTLA-4 levels are high [28]. Their data suggest that CD80-driven CD28 costimulation is more effectively opposed in the presence of abundant CTLA-4, whereas CD86 remains the more relevant ligand for sustaining the regulatory compartment [28]. These findings support the view that CD86 is a particularly important physiological target of CTLA-4 and that efficient receptor recycling is central to pathway function [27,28].

### 2.7. CTLA-4/PD-L1 Crosstalk via CD80

An important extension of CTLA-4 biology is its functional crosstalk with the PD-1/PD-L1 axis through CD80. Foundational work by Sugiura et al. showed that CD80 and PD-L1 interact in cis on the same antigen-presenting cell and that this association restricts PD-L1 binding to PD-1, thereby limiting PD-1 function during T-cell priming [29]. Zhao et al. further showed that the PD-L1:CD80 cis-heterodimer preserves CD80:CD28 costimulatory activity while repressing the PD-1 pathway and reducing CTLA-4 access to cis-complexed CD80 [30]. Together, these studies established CD80 as a point of physical and functional intersection between the two checkpoint systems.

Building on this foundation, Kennedy et al. showed that CTLA-4 can still bind and trans-endocytose CD80 even when CD80 is associated with PD-L1 [31]. This process is highly selective: CD80 is internalized and depleted, whereas PD-L1 remains on the donor-cell membrane [31]. As CD80 is progressively removed, free PD-L1 is restored in a time-dependent manner that correlates with the extent of CD80 removal [31]. In a three-cell functional assay, PD-L1 liberated by trans-endocytosis engaged PD-1 on responder T cells and inhibited TCR-driven CD69 upregulation, an effect reversed by PD-1 blockade, indicating that CTLA-4-mediated CD80 depletion can positively regulate the PD-1 pathway [31].

A key mechanistic point is that simple CTLA-4 binding is not sufficient to generate this effect. Kennedy et al. found that efficient liberation of PD-L1 required an intact CTLA-4 cytoplasmic domain and effective trans-endocytosis, whereas a tailless CTLA-4 mutant capable of limited trogocytosis was much less effective at restoring PD-1 binding [31]. Kennedy et al. also showed that soluble CTLA-4-Ig enhanced PD-L1 detection by anti-PD-L1 antibodies but did not restore PD-1-Ig binding [31]. Robinson et al. subsequently explained this discrepancy: soluble CTLA-4-Ig can only partially release PD-L1 when CD80 and PD-L1 are expressed at approximately equimolar levels, but fails when CD80 is in excess [32].

Robinson et al. further showed that release of PD-L1 from the CD80/PD-L1 cis heterodimer depends on the geometry of CTLA-4 engagement [32]. In their model, rigid, bivalent CTLA-4 binding is required to orient CD80 in a conformation that becomes unfavorable for renewed PD-L1 association, whereas monovalent or overly flexible CTLA-4 constructs fail to do so [32]. Notably, CD28-Ig, despite sharing the same MYPPPY-dependent binding site on CD80, also failed entirely to release PD-L1, consistent with its functionally monovalent mode of binding [32]. Even rigid, bivalent soluble CTLA-4 could release PD-L1 only under permissive CD80:PD-L1 ratios, whereas cell-expressed wild-type CTLA-4, by continually depleting CD80 through trans-endocytosis, restored PD-L1 availability regardless of CD80:PD-L1 expression ratios [32].

These data indicate that CTLA-4 is not only a regulator of CD28-mediated costimulation, but also an indirect regulator of PD-L1:PD-1 inhibition through its control of CD80 [29,30,31,32]. In this setting, CD80 becomes a point of crosstalk between the two checkpoint systems: when present in cis with PD-L1, this restrains PD-1 signaling; when removed by CTLA-4, this allows PD-L1 to engage PD-1 again. This has implications for combination immunotherapy: anti-CTLA-4 blockade would be expected to increase CD80 availability on APCs by inhibiting trans-endocytosis, thereby favoring CD80:PD-L1 cis interactions and potentially limiting PD-L1-mediated PD-1 signaling [31,32].

### 2.8. Soluble CTLA-4: An Unresolved Branch of CTLA-4 Biology

In addition to the canonical membrane receptor, CTLA-4 exists as a soluble isoform (sCTLA-4) generated by alternative splicing. The original description by Oaks et al. showed that alternative CTLA-4 transcripts lacking the transmembrane-encoding exon 3 are expressed in human, mouse, and rat hematolymphoid tissues, yielding a soluble form that preserves the extracellular ligand-binding domain but lacks membrane anchoring [33]. Because this splicing event removes the transmembrane segment and alters the downstream reading frame, the resulting isoform is structurally distinct from membrane CTLA-4 and cannot simply be considered a shed version of the full-length receptor [33]. Subsequent human studies and reviews further supported this basic exon organization and reinforced the concept that sCTLA-4 is a bona fide alternatively spliced product rather than a degradation fragment [34,35].

sCTLA-4 expression appears to be dynamically regulated rather than constitutive. Oaks et al. showed that the soluble transcript predominates in resting peripheral blood mononuclear cells, whereas activation shifts expression toward the full-length transmembrane isoform; Pawlak et al. likewise detected sCTLA-4 transcripts in resting human T-cell subsets and observed activation-related changes in abundance [33,34]. Human studies have also reported detectable circulating sCTLA-4, often at higher levels in autoimmune disease than in health, but its biological significance remains uncertain [34,35]. At present, sCTLA-4 is best viewed as a biologically plausible but still unresolved branch of CTLA-4 biology, rather than as an established determinant of human disease.

Functional studies suggest that sCTLA-4 is not merely an inert biomarker. In human systems, circulating or patient-derived sCTLA-4 can modulate T-cell proliferation and cytokine production, indicating biological activity, but these observations do not resolve whether its net role in vivo is protective, pathogenic, compensatory, or context-dependent [36,37].

More decisive mechanistic evidence comes from mouse models. Isoform-selective perturbation in NOD mice showed that reduced sCTLA-4 can impair Treg-mediated control of costimulation and accelerate autoimmunity in a sensitized background [38]. More recently, Osaki and Sakaguchi demonstrated in genetically engineered mice that soluble and membrane CTLA-4 are not functionally equivalent: selective loss of sCTLA-4 altered immune polarization and inflammatory-serologic homeostasis, whereas membrane CTLA-4 remained indispensable for preventing severe systemic autoimmunity [39].

These findings further support the biological plausibility of sCTLA-4, but also underline a major limitation: the strongest evidence for a non-redundant role of sCTLA-4 remains predominantly murine [39].

Current evidence supports three broad conclusions. First, sCTLA-4 is a genuine alternatively spliced isoform with distinct structural and regulatory properties [33,34,35]. Second, it has immunomodulatory activity, supported by both human in vitro studies and mouse genetic models [33,36,37,38,39]. Third, its role in human disease remains uncertain. In humans, the available evidence supports possible relevance through association studies and ex vivo functional observations, but does not yet establish a definite in vivo role in disease pathogenesis. At present, the strongest causal and mechanistic evidence for sCTLA-4 function remains predominantly murine. These molecular features do not operate uniformly across the immune system, making cell type the next essential level of interpretation.

## 3. Cell-Type-Specific Deployment of CTLA-4

### 3.1. Regulatory T Cells as the Dominant CTLA-4-Dependent Compartment

Among immune cell populations, regulatory T cells (Treg) represent the compartment most consistently and strongly dependent on CTLA-4 for physiological immune restraint. This conclusion rests first on genetic evidence. In the seminal BALB/c study by Wing et al., Treg-specific deletion of CTLA-4 caused fatal lymphoproliferative autoimmunity with multiorgan inflammation, including gastritis and cardiac inflammatory infiltrates, together with markedly increased serum IgE, establishing that CTLA-4 is indispensable for dominant Treg-mediated control in vivo [40]. Importantly, CTLA-4 deficiency did not prevent Treg development, survival, or lineage maintenance, indicating that CTLA-4 is required primarily for function rather than identity [40]. Jain et al. refined this interpretation by showing that CTLA-4-deficient Treg are not simply inert: they retain some CTLA-4-independent residual suppressive capacity, but cannot adequately prevent naïve T-cell activation, whereas CTLA-4 expression in activated conventional T cells mainly limits tissue infiltration and end-organ damage [41]. Treg cells appear to be the compartment most strongly dependent on CTLA-4, even though CTLA-4 function is not limited to them alone.

The main effector mechanism of Treg CTLA-4 is best understood as control of CD80/CD86 availability on antigen-presenting cells. Wing et al. showed that wild-type, but not CTLA-4-deficient, Treg downregulate CD80/CD86 on dendritic cells without affecting CD40 or MHC class II [40]. Qureshi et al. later provided the molecular basis for this observation by demonstrating trans-endocytosis of CD80/CD86 by CTLA-4 [23]. Ovcinnikovs et al. then clarified the in vivo hierarchy: Treg constitutively recruit cycling CTLA-4 to the surface ex vivo and perform ligand capture within hours of stimulation, whereas conventional T cells require prolonged activation before comparable activity becomes detectable [25]. In competitive in vivo settings, trans-endocytosis was therefore largely restricted to Treg, particularly ICOS+ Treg, and preferentially targeted migratory dendritic cells responding to self-antigen [25].

A critical interpretive point is that standard in vitro suppression assays can underestimate CTLA-4-dependent Treg function. Hou et al. showed that CTLA-4-dependent suppression is most evident in ligand-driven settings and varies with APC abundance and residual B7 levels, whereas anti-CD3/CD28 bead assays bypass the relevant ligand biology and fail to reveal CTLA-4-dependent inhibition [24]. Human evidence remains less direct than in mice but points in the same direction: Birebent et al. showed that sorting human CD4+CD25+ cells for surface CTLA-4/CD152 identified a more FOXP3-enriched subset, with 52-fold enrichment over PBMC versus 8-fold in the parallel CD152− fraction, and substantially stronger suppression of alloresponses (88% versus 58% inhibition), with partial reversal by anti-CTLA-4 blockade [10]. Direct human in vivo evidence for CTLA-4-dependent trans-endocytosis is still lacking. Thus, the strongest evidence for Treg dominance comes from mouse genetics and mechanistic murine studies, while the human literature currently provides supportive ex vivo rather than direct in vivo evidence. The same dependence on CTLA-4 is also evident in specialized regulatory populations that control humoral responses, particularly Tfr cells.

### 3.2. Follicular Regulatory T Cells and Germinal Center Control

T follicular regulatory (Tfr) cells are Foxp3+ CXCR5+ PD-1+ CD4+ cells that arise from thymic Foxp3+ precursors and acquire follicular features required for germinal center (GC) access, including dependence on Bcl-6, SAP, CD28, and interactions with B cells [42,43]. They therefore share features with both conventional Treg and Tfh cells, yet remain developmentally and functionally distinct from either lineage [42,43]. Experiments using CXCR5-deficient Foxp3+ cells showed that follicular localization is required for effective GC regulation, establishing Tfr cells as a specialized regulatory arm of the humoral response [43].

CTLA-4 is central to the suppressive function of this compartment. In Treg-specific CTLA-4 conditional knockout mice, Tfr cells are still generated, but they fail to restrain Tfh expansion, GC formation, and antigen-specific antibody responses, indicating that CTLA-4 is the dominant effector mechanism used by this compartment [44]. Sage et al. further showed that CTLA-4 deletion in differentiated Tfr cells substantially reduces their suppressive capacity while leaving Foxp3 expression intact, indicating a functional rather than developmental defect [45]. Mechanistically, non-follicular Treg and/or early Tfr can downregulate CD80/CD86, especially CD86, on B cells outside the GC, thereby limiting Tfh generation, whereas within the GC Tfr cells retain potent CTLA-4-dependent suppressive activity through a mechanism not explained simply by altered B7 expression [44,45].

A further layer of specialization is provided by CD25− Tfr cells, which preferentially localize within GCs, retain CTLA-4 expression and suppressive function, and have been identified in both murine and human lymphoid tissues [46]. Thus, CTLA-4-dependent Tfr biology provides a mechanistic bridge between checkpoint control and humoral tolerance: when this pathway fails, GC reactions expand, Tfh cells accumulate, and B-cell selection shifts toward excessive or autoreactive responses, helping explain the autoantibody formation, lymphoid hyperplasia, and humoral dysregulation seen in CTLA-4 pathway disease.

### 3.3. Conventional CD4 T Cells: Inducible Expression and Dual Roles

Unlike Treg cells, which constitutively express CTLA-4, conventional CD4+ T cells upregulate the receptor only after activation. In human peripheral blood, stimulation of CD4+CD25− cells generates a distinct CTLA-4+FOXP3+ subpopulation, and forced expression of CTLA-4, but not FOXP3 alone, is sufficient to confer suppressive activity, indicating that CTLA-4 itself can function as an inducible effector molecule in activated conventional CD4 T cells [11].

The clearest in vivo evidence for a cell-intrinsic role comes from the CT4Act model, in which CTLA-4 is expressed in activated conventional T cells but absent from Treg cells. In this setting, CTLA-4 on conventional T cells does not prevent the lymphoproliferation caused by CTLA-4-deficient Treg cells, but it markedly restrains the accumulation of activated T cells in non-lymphoid tissues and extends survival well beyond the usual 3–4 weeks of complete CTLA-4 deficiency [41]. These findings support a cell-intrinsic role for CTLA-4 in limiting the migratory pathogenicity of activated conventional T cells [41].

Conventional CD4 T cells can also use CTLA-4 in a cell-extrinsic manner. Two companion studies showed that cotransfer of CTLA-4-sufficient, Foxp3-negative conventional T cells normalized the excessive expansion of CTLA-4-deficient T cells responding either to tissue-expressed self-antigen or to foreign antigen [47,48]. Thus, activated conventional T cells can suppress neighboring T-cell responses in trans, likely by restricting access to shared costimulatory ligands. However, this activity is insufficient to prevent fatal systemic disease, confirming that Treg-expressed CTLA-4 remains the dominant mechanism of global immune restraint [41,47,48].

### 3.4. CD8 T Cells: Lower Expression, Selective Inhibitory Functions

Compared with CD4+ T cells and Treg cells, CTLA-4 appears to be a less dominant checkpoint in the CD8 compartment. In human peripheral blood, CTLA-4 mRNA and protein are induced less efficiently in activated CD8+ than CD4+ T cells, a difference associated with lower NFAT1 abundance and reduced histone acetylation at the proximal CTLA-4 promoter [12]. Under the conditions tested, anti-CTLA-4 blockade augments proliferation of human CD4+ but not CD8+ T cells [12]. Thus, in humans, CTLA-4 expression is inducible in CD8+ cells but quantitatively lower than in CD4+ cells, and the available functional evidence is limited to the experimental conditions tested in vitro [12].

Most mechanistic insight therefore comes from murine models. In mice, CTLA-4 can exert selective cell-intrinsic restraint on CD8 effector differentiation, cytotoxicity, and terminal maturation [49,50,51], although these effects can be masked by cell-extrinsic compensation, as suggested by mixed bone marrow chimera experiments and Treg-depletion settings [52,53]. Overall, CTLA-4 in CD8 T cells is best viewed as a context-dependent inhibitory mechanism whose importance is less directly established in humans than in the Treg compartment.

### 3.5. Human Evidence Versus Mouse-Based Inference

The strongest direct human evidence for cell-type-specific CTLA-4 function concerns the Treg compartment. In human CD4+CD25− T cells, enforced CTLA-4 expression is sufficient to confer suppressive activity, whereas FOXP3 expression alone is not, indicating that CTLA-4 can act as a functional effector of suppression in this setting [11]. In patients with LRBA deficiency, CTLA-4 protein is reduced in both FOXP3+ Treg cells and activated conventional T cells despite preserved mRNA, directly linking defective intracellular trafficking to human immune dysregulation [19]. Larger clinical cohorts have confirmed reduced Treg numbers, low CTLA-4 expression, and impaired suppressive function across LRBA deficiency and CTLA-4 haploinsufficiency [54]. Moreover, trans-endocytosis of CD80/CD86 by activated human CD4+ T cells and by human Treg cells has been demonstrated in vitro, including blockade by anti-CTLA-4 antibody and loss of function in patient-derived cells [19,23]. However, direct human in vivo evidence for trans-endocytosis remains lacking.

Beyond Treg cells, human data rapidly become thinner. Human Tfr cells have been identified phenotypically in tonsil and blood and retain CTLA-4 expression, but their CTLA-4-dependent suppressive function has been dissected almost entirely in mouse models [44,45,46]. For conventional CD4+ T cells, human studies show inducible CTLA-4 expression and preferential effects of CTLA-4 blockade on CD4 rather than CD8 proliferation [11,12], but the distinction between cell-intrinsic and cell-extrinsic roles in this compartment still rests mainly on murine transfer and conditional knockout systems [41,47,48]. The gap is even wider for CD8+ T cells: in humans, CTLA-4 is expressed at lower levels than in CD4+ cells and, under the conditions tested, may not measurably augment CD8+ T-cell proliferation when blocked in vitro [12], whereas the mechanistic framework for CD8-intrinsic CTLA-4 biology derives almost entirely from mouse studies [49,50,51].

This asymmetry in the evidence base requires caution when translating murine cell-type hierarchies directly into the interpretation of human CTLA-4 pathway disease. These cell-type differences become clinically visible when the pathway is disrupted by inherited defects, common variation, or therapeutic manipulation.

## 4. CTLA-4 Pathway Dysregulation Across Human Disease

### 4.1. Inborn Errors of Immunity as Natural Models of CTLA-4 Pathway Failure

Section 4 is organized into two parts. Section 4.1 and Section 4.2 address monogenic CTLA-4 pathway disorders, whereas Section 4.3, Section 4.4, Section 4.5 and Section 4.6 examine acquired, polygenic, pharmacologic, and therapeutic dysregulation of the same pathway. Within the monogenic group, CTLA-4 haploinsufficiency, LRBA deficiency, and DEF6 deficiency represent convergent inborn errors of immunity because all three reduce the surface availability of functional CTLA-4 on regulatory and activated T cells, thereby weakening a non-redundant checkpoint of peripheral tolerance [19,21,22,55,56,57,58]. Although they differ in their proximal molecular lesion, they converge on a shared biological consequence: impaired control of CD80/CD86-dependent costimulation, defective immune restraint, and a combined syndrome of lymphoproliferation, autoimmunity, humoral dysregulation, and susceptibility to infection [19,21,22,55,56,57,58]. As emphasized by Gámez-Díaz and Seidel, these disorders are best viewed not as isolated diseases, but as failures at different nodes of a common CTLA-4 control pathway [22]. Their shared mechanistic and clinical features are summarized in Table 1.

In CTLA-4 haploinsufficiency, heterozygous germline mutations directly reduce CTLA-4 dosage or function. Founding and subsequent cohorts showed reduced CTLA-4 expression and identified multiple mechanistic classes of pathway failure, including nonsense-mediated decay, defective ligand binding, impaired structural stability, and defective trans-endocytosis, all converging on reduced functional CTLA-4 at the cell surface [22,55,56,57].

In LRBA deficiency, CTLA-4 transcription is preserved but post-translational trafficking is impaired. LRBA colocalizes with CTLA-4 in intracellular vesicles, and LRBA-deficient patient T cells show markedly reduced total and surface CTLA-4 that can be partly rescued by chloroquine, indicating defective protection from lysosomal degradation and impaired recycling [19,22]. LRBA deficiency therefore represents the clearest human example of a trafficking/recycling defect in the CTLA-4 pathway [19,22].

DEF6 deficiency affects the same broader pathway through impaired RAB11-dependent recycling. Mutant DEF6 disrupts interaction with RAB11, reduces RAB11+CTLA-4+ vesicles, and impairs CTLA-4 surface trafficking; although only a few patients have been reported, the phenotype substantially overlaps with LRBA deficiency, with possible severe cardiac involvement in some cases [21,22].

These mechanistic differences are mirrored by both shared and divergent immunological phenotypes. In CTLA-4 haploinsufficiency, Treg cells are often preserved numerically but show reduced FOXP3/CD25 expression and impaired suppressive function, whereas effector T cells are hyperproliferative, illustrating the pathway’s dosage sensitivity [55]. B-cell abnormalities include progressive B-cell loss, expansion of CD21low autoreactive B cells, and hypogammaglobulinemia [55,57]. LRBA deficiency shows a closely related phenotype, although Treg counts appear more often reduced than in CTLA-4 haploinsufficiency [19,22,58].

Clinically, these disorders cluster around lymphoproliferation, autoimmunity, organ-specific inflammatory infiltration, and humoral immune failure. In CTLA-4 haploinsufficiency, the largest cohort reported frequent hypogammaglobulinemia, lymphoproliferation, respiratory and gastrointestinal disease, autoimmune cytopenia, and neurologic involvement, with marked variable expressivity, incomplete penetrance, and non-trivial mortality [57]. Comparative analyses suggest that penetrance estimates vary with cohort composition and ascertainment, ranging from 60.6% in systematic aggregation to 67.6% (90/133) in the largest clinically referred cohort [57,58,59]. Malignancy, particularly lymphoma, is also part of the phenotype spectrum and may in some cases be linked to EBV-associated immune dysregulation [57].

The systematic comparison by Jamee et al. clarified differences between CTLA-4 haploinsufficiency and LRBA deficiency: LRBA deficiency presents much earlier (median onset 1.7 vs. 10.0 years), is more strongly associated with consanguinity, and tends to show more autoimmune enteropathy, organomegaly, and growth failure, whereas CTLA-4 haploinsufficiency shows more granulomatous disease, neurologic manifestations, and malignancy [58]. LRBA deficiency also appears more consistently penetrant, in contrast to the clearly incomplete penetrance of CTLA-4 haploinsufficiency [22,58]. Nonetheless, the overlap is striking enough that both disorders fit within a unified concept of CTLA-4 pathway failure rather than separate mechanistic universes [19,22,58].

Together, these disorders show that the CTLA-4 pathway can fail through structural defects in CTLA-4 itself, through loss of ligand handling/trans-endocytosis, or through defective intracellular recycling and trafficking. Their strong phenotypic overlap argues that what matters clinically is not the precise mutated gene alone, but the final common outcome: inadequate functional CTLA-4 at the cell surface. This makes these inborn errors of immunity the clearest natural models for understanding how disruption of CTLA-4 biology translates into human disease.

### 4.2. Clinical Heterogeneity in CTLA-4 Pathway Inborn Errors

Despite their convergence on a shared molecular endpoint—reduced surface availability of functional CTLA-4–CTLA-4 pathway inborn errors show striking heterogeneity in penetrance, age at onset, organ tropism, and severity. This is clearest in CTLA-4 haploinsufficiency, where penetrance ranges from 60.6% in family-based systematic aggregation [58] to 67.6% (90/133) in the largest single cohort [57]. The latter figure refers to affected individuals among mutation carriers in a clinically referred cohort, whereas the lower estimate derives from family-based aggregation; the two values therefore reflect different ascertainment strategies and are not directly interchangeable. Thus, roughly one-third of mutation carriers may remain clinically unaffected. Crucially, Schubert et al. showed that asymptomatic carriers can display the same reduction in CTLA-4 expression, trans-endocytosis, and Treg suppressive function as affected relatives, suggesting that the core biochemical defect is not sufficient by itself to determine clinical disease [56]. Kuehn et al. likewise described marked intrafamilial variability, including clinically unaffected carriers of the same mutation found in severely affected relatives [55].

By contrast, LRBA deficiency appears to have nearly complete penetrance overall, although rare asymptomatic siblings have been reported [58,59,60]. It also presents much earlier, with a median onset of 1.7 years; in comparative analyses, the corresponding median onset for CTLA-4 haploinsufficiency is approximately 10.0 years, while the largest cohort-based CTLA-4 insufficiency study reported a median age at onset of 11 years [57,58]. Organ-specific phenotypes diverge in ways not fully predicted by the shared pathway defect. Jamee et al. showed that granulomatous disease is more frequent in CTLA-4 haploinsufficiency (CHAI) (*p* < 0.001), whereas autoimmune enteropathy (*p* = 0.038) and organomegaly (*p* = 0.023) are more prominent in LRBA deficiency [58]. Barış further highlighted that GLILD is much more frequent in CTLA-4 insufficiency (36% vs. 6.8%), as are neurologic involvement and malignancy (12.9–17.4%) [59]. Immunologically, Treg counts are often normal in CHAI but more often reduced in LRBA deficiency [58]. No robust genotype–phenotype correlation has been established in either disorder [56,57,58].

The basis of this heterogeneity remains only partly understood. Residual protein expression likely matters: patients with some preserved LRBA expression retain higher CTLA-4 levels, suggesting a dose effect [19]. Environmental triggers may also contribute; in CTLA-4 haploinsufficiency, EBV infection was clinically apparent in 16 of 90 affected carriers, and EBV positivity was reported in 5 of 8 lymphomas in supplementary cohort-level data presented with the study [57]. Multiple studies therefore invoke modifier genes, epigenetic mechanisms, and infectious or environmental triggers, but none has yet been firmly identified [55,56,58,59]. Molecular diagnosis identifies the pathway defect, but it still does not predict the individual clinical trajectory.

### 4.3. Polygenic Autoimmunity: Quantitative CTLA-4 Dysfunction

The monogenic disorders discussed in Section 4.1 and Section 4.2 show that major loss of CTLA-4 pathway function can produce broad, early-onset immune dysregulation. By contrast, polygenic autoimmunity is better understood as a state of quantitative CTLA-4 pathway dysfunction: inherited variation that modestly alters pathway output without causing the near-structural failure seen in CTLA-4 haploinsufficiency, LRBA deficiency, or DEF6 deficiency.

The strongest evidence comes from common variation at the CTLA-4 locus. Ueda et al. mapped susceptibility to autoimmune disease to a non-coding 6.1-kb region 3′ of CTLA-4, identifying CT60 (rs3087243) as the most strongly associated marker. In Graves disease, CT60 conferred an odds ratio of 1.51 (95% CI 1.31–1.75; *p* = 2.72 × 10^−8^), whereas the effect in type 1 diabetes was smaller (OR < 1.15) [63]. This contrast is informative in itself: common CTLA-4 alleles exert relatively small effects, consistent with pathway modulation rather than categorical pathway failure. Subsequent association studies and meta-analyses have broadly supported the contribution of CT60 and +49A/G (rs231775) to common autoimmune disease, particularly autoimmune thyroid disease and type 1 diabetes, with additional signals reported in other autoimmune settings [64,65,66,67].

The proposed mechanistic link between these common variants and disease has centered on isoform regulation, especially soluble CTLA-4 (sCTLA-4). Ueda et al. reported that the ratio of sCTLA-4 to full-length CTLA-4 mRNA in unstimulated human CD4 T cells was approximately 50% lower in susceptible CT60 G/G homozygotes than in protective A/A individuals, with a semidominant pattern consistent with dosage sensitivity [63]. However, this interpretation remains unresolved. Not all subsequent studies replicated a strong allelic effect of CT60 on splicing, and the functional consequences may extend beyond altered transcript balance alone, potentially including changes in T-cell signaling [64,68]. Thus, the genetic association is robust, but the precise molecular mechanism remains uncertain.

The functional significance of sCTLA-4 has been explored more directly than its genetic regulation, although much of the mechanistic work remains non-human. Early work identified sCTLA-4 as a naturally generated splice product with measurable immunomodulatory activity in vitro [33]. In a murine NOD model, Gerold et al. selectively silenced sCTLA-4 while preserving other CTLA-4 isoforms and showed impaired Treg suppressive function, reduced inhibition of dendritic-cell CD86, failure to control colitis, and accelerated diabetes onset on the protected Idd5.1 background [38]. In humans, elevated circulating sCTLA-4 has been reported in several autoimmune diseases, and patient-derived sCTLA-4 can modulate T-cell proliferation and cytokine production in vitro [36]. These observations support possible human relevance, but they remain associative or ex vivo and do not by themselves establish a defined in vivo pathogenic role in human disease. Whether the observed signal reflects a pathogenic mechanism, a compensatory response, or simply a biomarker of immune activation therefore remains unclear.

This contrast helps clarify the difference between monogenic and polygenic CTLA-4 dysfunction. CTLA-4 haploinsufficiency produces incompletely penetrant but often multisystem immune dysregulation, whereas common CTLA-4 variants exert modest effect sizes and are associated mainly with organ-specific autoimmune phenotypes. This supports a dosage-threshold model: small inherited reductions in CTLA-4 pathway output may not be sufficient to cause disease on their own, but can shift the balance toward autoimmunity when combined with other genetic and environmental risk factors.

### 4.4. Transplantation: When Costimulation Blockade Also Impairs Coinhibition

In kidney transplantation, belatacept is the best-established clinical example of CTLA-4 pathway agonism. The BENEFIT program showed that costimulation blockade with belatacept preserves superior long-term renal function and reduces the composite risk of death or graft loss compared with cyclosporine-based regimens, albeit at the cost of increased early acute rejection—a paradox that may partly reflect the fact that B7 blockade also limits endogenous CTLA-4 coinhibition on effector and regulatory T cells [69,70,71,72,73,74,75,76,77]. Selective CD28 blockade, which preserves endogenous CTLA-4 signaling, has therefore emerged as a strategy to overcome this limitation (see Section 5.2 for detailed trial outcomes, donor-specific antibody formation, and long-term follow-up) [75,76,77].

### 4.5. Cancer Immunotherapy: Intentional Antagonism of the CTLA-4 Pathway

Because endogenous CTLA-4 helps preserve peripheral tolerance by limiting costimulation and sustaining regulatory control, anti-CTLA-4 cancer immunotherapy can be viewed as a deliberate disruption of a physiological tolerance pathway. Clinically, this strategy can produce durable responses in a subset of patients, but the underlying biology is more complex than simple “checkpoint release”. Available structural, mechanistic, and translational data indicate that anti-CTLA-4 antibodies act through at least two partially overlapping mechanisms: steric blockade of CTLA-4:B7 interactions and Fc gamma receptor (FcγR)-dependent depletion of intratumoral Treg cells [7,78,79,80]. However, the evidence base is not symmetrical: steric blockade is supported by structural and translational data, whereas the strongest direct evidence for FcγR-dependent intratumoral Treg depletion remains preclinical and predominantly murine [7,78,79,80].

The structural basis for blockade is clear. Ramagopal et al. showed that ipilimumab binds the front β-sheet of CTLA-4 and directly overlaps the B7-binding surface, providing a straightforward explanation for competitive inhibition of ligand binding [7]. Yet blockade alone does not fully explain therapeutic efficacy. In human FcγR knock-in models, Arce Vargas et al. showed that Fc-silent IgG1-N297A anti-CTLA-4 antibodies retained ligand-blocking activity but failed to promote tumor rejection, indicating that CTLA-4 antagonism without Fc effector function is insufficient in that setting [79].

A large body of preclinical work supports intratumoral Treg depletion as a major determinant of antitumor activity. Simpson et al. first showed that anti-CTLA-4 selectively depletes Treg cells within tumors but not in draining lymph nodes, and that this effect depends on FcγRIV-expressing macrophages [78]. Importantly, depletion required direct antibody–CTLA-4 interaction rather than functional blockade alone, because CTLA-4-deficient Treg cells were not eliminated [78]. The spatial specificity of this mechanism is biologically important: CD11b+ FcγRIV+ myeloid cells were enriched roughly 70-fold in tumors compared with lymph nodes, and intratumoral Treg cells displayed greater accessible CTLA-4 than their peripheral counterparts [78]. Arce Vargas et al. extended this logic toward humans, showing that both human IgG1- and IgG2-formatted anti-CTLA-4 antibodies can deplete intratumoral Treg cells, while Fc-enhanced IgG1-SDALIE achieved the strongest tumor control [79]. Thus, in humans, support for this mechanism remains indirect rather than based on direct quantitative demonstration of intratumoral Treg depletion in vivo [79,81]. Notably, even depleting isotypes failed in poorly infiltrated B16 tumors, indicating that Fc-mediated efficacy still depends on a permissive tumor microenvironment [79].

These observations are best reconciled by viewing Fc-dependent intratumoral Treg depletion as a major but not sufficient component of anti-CTLA-4 efficacy. In other words, maximal antitumor activity appears to require dual function: depletion of intratumoral Treg cells together with checkpoint antagonism. Consistent with this, Lax et al. used a non-antagonistic CTLA-4 binder that depleted intratumoral Treg cells as effectively as 9d9 but did not block B7 binding or interfere with trans-endocytosis [80]. Although this construct improved survival, only 9d9—which combines antagonism plus depletion—produced the highest cure rate, with intratumoral dosing yielding 47% versus 0% cure [80]. Anti-CTLA-4 also has distinct effects on effector programs: Wei et al. showed by mass cytometry that anti-CTLA-4, but not anti-PD-1, specifically expands an ICOS+ TBET+ Th1-like CD4 effector subset, a pattern reproduced across tumor models and confirmed in human melanoma samples [81]. Thus, anti-CTLA-4 therapy is not merely subtractive; it both removes regulatory barriers and reshapes intratumoral effector immunity.

This broader mechanistic picture also helps connect CTLA-4 biology to the rationale for combined PD-1/CTLA-4 blockade. As discussed in Section 2.7, CTLA-4-mediated trans-endocytosis of CD80 can liberate PD-L1 from CD80:PD-L1 cis heterodimers, thereby restoring PD-L1 availability for PD-1 engagement [29,30,31,32]. Conversely, anti-CTLA-4 blockade would be expected to interrupt this route of PD-L1 liberation, while simultaneously increasing costimulatory pressure and reshaping the intratumoral effector/regulatory balance through checkpoint antagonism and Fc-dependent Treg depletion [29,30,31,32,78,79,80,81]. The net effect on PD-L1 availability is therefore likely to be context-dependent, but this crosstalk provides one plausible mechanistic basis for why PD-1 blockade can complement anti-CTLA-4 therapy by neutralizing a distinct, intersecting inhibitory pathway [29,30,31,32]. This same mechanistic framework helps explain why Fc engineering and endosomal trafficking have become central to next-generation anti-CTLA-4 design. Botensilimab, an Fc-enhanced anti-CTLA-4 antibody, showed activity in poorly immunogenic and immune checkpoint inhibitor (ICI)-refractory tumors and appeared less dependent on host *FCGR3A* genotype, consistent with stronger myeloid engagement and more efficient Treg depletion [82]. Conversely, Zhang et al. showed that conventional antibodies such as ipilimumab can direct CTLA-4 toward lysosomal degradation, whereas pH-sensitive antibodies that dissociate after endocytosis preserve LRBA-dependent CTLA-4 recycling and improve the balance between antitumor efficacy and systemic toxicity [83]. Strikingly, disruption of CTLA-4 recycling through LRBA knockdown converted a normally non-irAE-prone antibody into a toxic phenotype, directly linking recycling failure to treatment-associated immune toxicity [83].

From this perspective, anti-CTLA-4 therapy is best seen as context-dependent pathway antagonism, used to generate preferential benefit within the tumor microenvironment. The consequences of that antagonism outside the tumor are addressed in Section 4.6.

### 4.6. Immune-Related Adverse Events as Pharmacologic Phenocopies of the CTLA-4 Pathway Deficiency

The immune-related adverse events (irAEs) that complicate anti-CTLA-4 cancer immunotherapy can, in many respects, be viewed as a pharmacologic form of CTLA-4 pathway insufficiency. In clinical terms, they recapitulate in compressed form many of the manifestations seen in monogenic CTLA-4 pathway disorders, including enterocolitis, hepatitis, autoimmune cytopenias, and multiorgan lymphocytic infiltration [84,85]. This overlap supports the idea that irAEs are not simply off-target toxicity, but an on-pathway consequence of reducing functional CTLA-4-mediated immune restraint [84,85].

The mechanistic bridge is now clearer. Zhang et al. showed that ipilimumab and TremeIgG1 rapidly direct surface CTLA-4 toward lysosomal degradation, whereas non-irAE-prone, pH-sensitive anti-CTLA-4 antibodies dissociate after endocytosis and preserve LRBA-dependent CTLA-4 recycling [83]. Disrupting recycling was sufficient to confer toxicity to an otherwise non-irAE-prone antibody, directly linking antibody-induced CTLA-4 degradation to irAE development [83]. This is a striking point of convergence with LRBA deficiency, in which genetic failure of CTLA-4 recycling causes immune dysregulation through the same trafficking axis. Preliminary immune profiling reported in a conference abstract suggests that patients developing clinically significant irAEs may display Treg perturbations resembling those described in CTLA-4 haploinsufficiency [86].

At the same time, pharmacologic and genetic CTLA-4 deficiency are not identical. The clearest divergence is hypophysitis, which appears to be an antibody-specific, on-target/off-tumor toxicity rather than a generic consequence of CTLA-4 insufficiency. Iwama et al. demonstrated that pituitary endocrine cells ectopically express CTLA-4, and that anti-CTLA-4 antibody administration induces local C3d/C4d complement deposition, consistent with a type II hypersensitivity mechanism [87]. Caturegli et al. confirmed this in human autopsy material, showing IgG-dependent complement fixation, CD68+ macrophage infiltration, and CD4+ T-cell-predominant lymphocytic inflammation with features of both type II and type IV hypersensitivity [88]. Consistent with this mechanism, hypophysitis occurs in approximately 4% of patients receiving ipilimumab monotherapy at 3 mg/kg (106/2853 patients across 21 clinical trials) [87], although later estimates that include higher doses and combination regimens with anti-PD-1 antibodies suggest rates approaching 10% [88]. The lower rates reported with tremelimumab are also consistent with an Fc-subclass-dependent mechanism [87]. No comparable pituitary phenotype is described in CHAI or LRBA deficiency, underscoring that some irAEs depend on the physical presence of an exogenous antibody rather than CTLA-4 deficiency alone. irAEs therefore provide a useful pharmacologic model for understanding CTLA-4 biology in humans. They show that transient therapeutic disruption of CTLA-4 is sufficient to reproduce many features of genetic pathway failure, while also revealing antibody-specific toxicities that genetic deficiency alone does not generate.

### 4.7. A Unified Cross-Disease Model of CTLA-4 Pathway Dysfunction

The disease settings reviewed in Section 4.1, Section 4.2, Section 4.3, Section 4.4, Section 4.5 and Section 4.6 appear clinically diverse, yet they converge on a single functional deficit: insufficient effective CTLA-4 activity at the T cell–APC interface, with inadequate control of CD80/CD86 ligands and peripheral tolerance [19,21,22,36,38,55,56,57,58,59,63,64,65,66,67,68,89]. This deficit can arise through multiple modes of perturbation. CTLA-4 haploinsufficiency reduces total protein dosage; LRBA and DEF6 deficiencies impair recycling and deplete the steady-state surface pool; common CTLA-4 variants impose smaller quantitative reductions in pathway output that contribute to polygenic autoimmunity [19,21,22,36,38,55,56,57,58,59,63,64,65,66,67,68,89]. In transplantation, CTLA-4-Ig blocks CD28 costimulation but also limits endogenous CTLA-4 access to B7 ligands, weakening coinhibition [69,70,71,72,73,74,75,76,77,90]. In oncology, anti-CTLA-4 antibodies deliberately antagonize the pathway and may additionally promote lysosomal CTLA-4 loss; the resulting irAEs therefore represent a pharmacologic phenocopy of genetic pathway failure [7,54,78,79,80,81,82,83,84,85,86,87,88,91,92,93].

What unifies these conditions is not the initiating insult but the downstream consequence: reduced functional CTLA-4 below the threshold required for stable immune regulation. Clinical severity then scales with the depth, duration, and context of this deficit, from lifelong high-penetrance autoimmunity in monogenic disease, through modest quantitative susceptibility in polygenic autoimmunity, to therapeutic pathway manipulation in transplantation and cancer. This suggests that CTLA-4-related pathology is better viewed as a spectrum of pathway insufficiency rather than as a set of discrete diseases, and that therapeutic targeting should be calibrated to where each patient lies on that spectrum (Section 5).

## 5. Therapeutic Targeting of the CTLA-4 Pathway

Taken together, the therapeutic targeting of the CTLA-4 pathway is context-dependent rather than unidirectional. In monogenic CTLA-4 pathway insufficiency, the goal is pathway replacement or restoration, as illustrated by the use of abatacept to compensate for impaired endogenous CTLA-4 function. In transplantation, the objective is not simply broader costimulatory blockade, but preservation of endogenous CTLA-4-mediated coinhibition, which provides a rationale for selective CD28 blockade over strategies that also prevent physiologic CTLA-4 access to CD80/CD86. In cancer immunotherapy, the desired effect is the opposite: preferential antagonism of CTLA-4 pathway output within the tumor microenvironment, ideally combined with Fc properties and trafficking behavior that maximize antitumor efficacy while limiting systemic toxicity. In immune-related adverse events, management instead seeks to restore immune restraint rather than intensify pathway blockade further. Across these settings, the key principle is that stronger intervention is not necessarily better; rather, the optimal strategy depends on whether the clinical problem is insufficient, excessive, or mislocalized CTLA-4 pathway output.

### 5.1. Abatacept: Pathway Replacement, Immunomodulation, and Translational Lessons

Abatacept is a soluble CTLA-4-Ig fusion protein composed of the extracellular domain of CTLA-4 linked to a modified IgG1 Fc region. By binding CD80/CD86 on antigen-presenting cells, it blocks CD28-mediated costimulation and functionally substitutes for one key output of endogenous CTLA-4 biology: ligand sequestration at the APC interface [19]. In CTLA-4 pathway disorders, its significance is therefore more specific than generic immunosuppression. In this setting, abatacept is better viewed as functional pathway replacement than as nonspecific immunosuppression, because it restores control over CD80/CD86 when endogenous CTLA-4 is quantitatively insufficient or functionally unavailable [19].

The rationale for abatacept in monogenic disease rests on convergence. CTLA-4 haploinsufficiency reduces receptor dosage, LRBA deficiency accelerates lysosomal loss of CTLA-4, and DEF6 deficiency disrupts RAB11-dependent trafficking; however, all converge on inadequate functional CTLA-4 at the cell surface [19,21]. Lo et al. showed that patients with LRBA deficiency had a dramatic and sustained response to abatacept, including marked improvement of lymphocytic interstitial lung disease and enteropathy with follow-up extending up to 7 years [19]. Serwas et al. then extended the same principle to DEF6 deficiency: one of the three reported patients achieved sustained remission of severe enteropathy [21]. The fact that abatacept works across different upstream defects supports the idea that it acts at a shared functional bottleneck—control of CD80/CD86—rather than at the level of the mutated gene itself [19,21].

The strongest organ-specific treatment data come from the Freiburg cohort reported by Egg et al. Among 173 CTLA-4 mutation carriers, 123 required treatment for immune complications and 29 received abatacept [61]. Responses were clearly organ dependent: GLILD improved in 7 of 10 patients (70%), gastrointestinal disease in 9 of 9, and cytopenia in 24 of 29 [61]. Improvement was also reported in selected central nervous system lymphocytic infiltrates, but relapse remained possible—especially in gastrointestinal disease—and three patients worsened on therapy, underscoring that pathway replacement is only partially restorative [61]. Egg et al. therefore proposed abatacept as a major steroid-sparing option, particularly for enteropathy and GLILD, while also launching the prospective ABACHAI trial to address optimal dosing, timing, and predictors of response [61,94].

Long-term comparative data were then provided by Taghizade Mortezaee et al. in a multicenter cohort of 98 patients with LRBA deficiency or CTLA-4 insufficiency [54]. Among 58 patients treated with abatacept, 46 (79.3%) achieved complete response, clearly outperforming conventional immunosuppressants used as primary therapy, for which complete response was observed in only 27.9% [54]. Nonresponders to abatacept had longer disease duration and broader organ involvement, suggesting that disease chronicity and accumulated tissue injury constrain reversibility [54]. In the same cohort, HSCT remained effective in selected severe cases, while the high complete-response rate observed with abatacept further supported its role as a genuine disease-modifying strategy rather than only a temporary bridge [54]. Taghizade Mortezaee et al. also suggested that sirolimus plus abatacept can be useful in difficult cases, highlighting that pathway replacement sometimes benefits from combination therapy when inflammatory burden is high [54,59].

Mechanistic correlates of response help explain why abatacept is so informative biologically. Charbonnier et al. showed that LRBA deficiency is associated with marked Treg impairment and broad autoantibody production [95]. Alroqi et al. then demonstrated exaggerated circulating Tfh responses together with reduced Tfr cells and showed that cTfh frequencies decline with CTLA-4-Ig therapy [96]. Taghizade Mortezaee et al. and Catak et al. extended these observations by showing that abatacept can rebalance naïve versus memory lymphocyte compartments while reducing activated cTfh cells and CD21low B cells, supporting the idea that follicular dysregulation is one of the treatment-responsive outputs of CTLA-4 insufficiency [54,96,97].

The most comprehensive translational dataset comes from Catak et al., who integrated longitudinal flow cytometry, NanoString transcriptomics (785 genes), SomaScan proteomics, and single-cell RNA sequencing in LRBA-deficient and CTLA-4-insufficient patients [97]. Before treatment, patients showed compensatory upregulation of alternative inhibitory checkpoints, including *LAG3*, *TIGIT*, *HAVCR2*, *ADORA2A*, and *VSIR*, together with broad inflammatory pathway activation [97]. Abatacept reversed much of this program, normalizing lymphoid signatures more strongly than myeloid ones and identifying *BCL2L1* as a potential biomarker of disease activity and response [97]. Importantly, the same study also showed a paradoxical limitation of therapy: baseline Treg deficiency was further exacerbated by CD28 blockade, illustrating that abatacept restores one core function of CTLA-4 while simultaneously failing to reproduce the full biology of endogenous CTLA-4-dependent Treg homeostasis [97]. Persistent type 1 diabetes in several patients further emphasized that abatacept can control active immune dysregulation more effectively than it can reverse established end-organ destruction [97].

These findings highlight both the strengths and the limitations of abatacept. It does not recreate the endogenous intracellular CTLA-4 reservoir, its regulated mobilization after TCR stimulation, or the full spatial and cell-type-specific deployment of physiological CTLA-4 function [19,97]. Yet its efficacy across CHAI, LRBA deficiency, and DEF6 deficiency demonstrates that insufficient CTLA-4 is itself therapeutically tractable in humans [19,21,54,61,97]. Abatacept is therefore best viewed not simply as a drug, but as a strong translational proof that CTLA-4 pathway supplementation can correct a convergent immunoregulatory defect across genetically distinct diseases [19,54,61,97]. The principal therapeutic modalities currently used in CTLA-4 pathway disorders are summarized in Table 2.

### 5.2. Belatacept and the Transplantation Paradox

Belatacept is a second-generation CTLA-4-Ig fusion protein developed for kidney transplantation to inhibit CD28-dependent costimulation through high-avidity binding to CD80/CD86 [69,77]. Mechanistically, however, it also creates a revealing paradox: by occupying B7 ligands, belatacept not only prevents CD28 signaling but also limits endogenous CTLA-4 access to the same ligands, thereby attenuating coinhibition at the same time that it suppresses costimulation [69,77].

The clinical dimensions of this paradox were defined by the BENEFIT program. In the original trial, belatacept improved renal function relative to cyclosporine at 12 months, with mean measured GFR values of 65, 63, and 50 mL/min/1.73 m^2^ in the more intensive belatacept, less intensive belatacept, and cyclosporine groups, respectively, while acute rejection rates at 12 months were higher (22%, 17%, and 7%) [69]. Long-term follow-up then showed that belatacept reduced the composite risk of death or graft loss by 43% compared with cyclosporine (HR 0.57, 95% CI 0.35–0.95), preserved a marked eGFR advantage at 7 years (70.4–72.1 vs. 44.9 mL/min/1.73 m^2^), and markedly lowered de novo donor-specific antibody formation (1.9–4.6% vs. 17.8%) [70]. In BENEFIT-EXT, belatacept similarly preserved renal function in extended-criteria donor recipients (53.9–54.2 vs. 35.3 mL/min/1.73 m^2^), although survival differences were not significant [71]. Thus, belatacept improves long-term graft quality yet incompletely controls early rejection.

This pattern likely reflects a central limitation of ligand blockade: B7 sequestration is not equivalent to physiologic CTLA-4 function. In humans, belatacept did not produce a major long-term depletion of circulating Tregs compared with CNI therapy, but rejecting grafts from belatacept-treated patients contained significantly more FOXP3+ cells, suggesting that regulatory-cell presence is not sufficient when endogenous CTLA-4-mediated ligand control is blocked [73]. In mice, CTLA-4-Ig reduced Treg numbers and preferentially affected Helios+ thymus-derived Tregs, accelerating rejection in a Treg-dependent transplant model [72]. In a head-to-head baboon kidney transplant study, belatacept caused severe steroid-resistant rejection in 4 of 5 animals, whereas the selective CD28 antagonist FR104 avoided severe steroid-resistant rejection and was associated with lower intragraft IL-21 and better preservation of regulatory signatures [74]. These findings explain why selective CD28 blockade emerged as the logical next step: unlike CTLA-4-Ig, it suppresses costimulation while preserving endogenous CTLA-4 coinhibition [74,77].

Belatacept therefore highlights an important aspect of CTLA-4 biology in humans: blocking B7 ligands can improve renal and humoral outcomes, but it is not the same as preserving the physiological CTLA-4 pathway. The transplantation paradox is that the same intervention that limits CD28 activation can simultaneously undermine the endogenous regulatory system that normally constrains alloresponses.

### 5.3. Ipilimumab and Tremelimumab: Checkpoint Blockade, Treg Depletion, and Fc Biology

First-generation anti-CTLA-4 antibodies are best viewed as acting through a dual mechanism: checkpoint antagonism together with Fc-mediated depletion of intratumoral Treg cells, rather than receptor blockade alone [7,78,79,80]. That view helps explain why ipilimumab and tremelimumab can produce durable antitumor responses despite substantial toxicity, and why Fc isotype and FcγR context shape efficacy in both preclinical models and translational datasets [79].

This mechanistic view has influenced the design of newer anti-CTLA-4 agents. Fc-enhanced backbones aim to strengthen tumor-localized depletion, whereas pH-sensitive and other context-selective formats aim to preserve CTLA-4 recycling and reduce peripheral toxicity without sacrificing intratumoral activity (see Section 5.4) [82,83,98,99,100,101]. In human translational datasets, FcγR-dependent mechanisms also appear relevant, but no FcγR-based biomarker has become clinically established. The broader challenge remains the same: to separate tumor efficacy from autoimmunity, given that systemic Treg depletion and global checkpoint blockade likely both contribute to irAEs [79,82,83,98,99,100,101].

### 5.4. Next-Generation Anti-CTLA-4 Strategies

Next-generation anti-CTLA-4 antibodies were developed in response to a clear problem revealed by the first generation: systemic checkpoint blockade is difficult to disentangle from systemic toxicity. As discussed in Section 5.3, first-generation agents—most clearly ipilimumab—can combine CTLA-4 antagonism with Fc-dependent intratumoral Treg depletion. Newer designs therefore aim not simply to block CTLA-4 more strongly, but to improve the balance between efficacy and toxicity by preserving tumor-localized benefit while reducing peripheral CTLA-4 loss and immune dysregulation [79,82,83,98]. Major design strategies and their mechanistic logic are summarized in Table 3.

A first major strategy is recycling-preserving, pH-sensitive antibody design. Zhang et al. showed that conventional antibodies such as ipilimumab, and an engineered IgG1 chimeric version of tremelimumab (TremeIgG1) used for mouse experiments, can drive CTLA-4 toward lysosomal degradation, whereas pH-sensitive antibodies dissociate after endocytosis and permit LRBA-dependent CTLA-4 recycling back to the cell surface [83]. This established an important biological point: preserving CTLA-4 recycling can improve both safety and antitumor efficacy, indicating that antibody-induced CTLA-4 degradation is not required for tumor control and may contribute substantially to treatment-associated toxicity. Zhang et al. then refined this principle by systematically engineering pH-sensitive variants of ipilimumab and tremelimumab and showing that the critical determinant is early-endosomal dissociation at pH 6.0, not merely weaker affinity or dissociation at later lysosomal pH [98]. Variants such as Ipi25 and Treme156/157 preserved surface CTLA-4 on Tregs, restored Rab11-dependent recycling, reduced anemia and multiorgan inflammation, and improved tumor control; importantly, the control variant Ipi14, which had similarly reduced affinity but lacked pH 6.0 sensitivity, failed to confer these advantages and even showed worse antitumor efficacy than parental ipilimumab [98]. These trafficking studies were confirmed not only in tumor models but also in human PBMC-derived Tregs, strengthening their translational relevance [98].

A second, mechanistically distinct strategy is tumor-acidic pH selectivity rather than endosomal dissociation. Lee et al. engineered anti-CTLA-4 variants with more than 50-fold stronger binding at pH 6.0 than at pH 7.4, thereby favoring activity within the acidic tumor microenvironment while limiting peripheral target engagement [99]. In human CTLA-4 knock-in mice, these pH-selective antibodies preserved antitumor efficacy and intratumoral Treg depletion at levels comparable to nonselective ipilimumab, while markedly reducing peripheral immune activation: notably, splenic Treg Ki-67 returned to near-isotype-control levels, whereas nonselective ipilimumab significantly increased peripheral Treg proliferation [99]. Gao et al. provided complementary structural support for this broader class of pH-sensitive anti-CTLA-4 antibodies by solving the HL32–CTLA-4 complex and showing how four histidines at the interface—one on CTLA-4 and three on HL32—confer pH-responsive binding, with an approximately 3.4-fold reduction in affinity from pH 7.4 to 5.5 [100]. These studies define two distinct pH strategies that converge on the same biological goal: one exploits acidic extracellular pH in the tumor, whereas the other uses acidic early endosomes to preserve recycling.

A third strategy is Fc enhancement, designed to amplify the beneficial effector arm of anti-CTLA-4 therapy within tumors. This is best interpreted in light of Lax et al., who showed that depletion alone is not enough: a nonantagonistic Treg-depleting anti-CTLA-4 construct improved survival but produced 0% cure, whereas the antagonistic and depleting 9d9 antibody achieved 47% cure with intratumoral dosing [80]. Thus, stronger Fc function is valuable not because “more depletion is always better,” but because it can potentiate the depletion arm while preserving the synergy with CTLA-4 antagonism. Chand et al. showed that the Fc-enhanced antibody botensilimab can achieve activity in poorly immunogenic and ICI-refractory tumors while appearing less dependent on host FCGR3A genotype than first-generation agents [82]. Blanchard et al. extended this concept by demonstrating that Fc optimization does more than intensify Treg depletion: Fc-competent anti-CTLA-4 also promotes the formation of tumor-associated high endothelial venules, in a mechanism dependent on both Fc effector function and CD4+ T cells, thereby potentially improving lymphocyte recruitment into otherwise refractory tumors [101].

Next-generation anti-CTLA-4 strategies point to three main lessons. First, stronger global CTLA-4 antagonism is not necessarily better [80,98]. Second, preserving CTLA-4 recycling may improve both safety and efficacy, suggesting that lysosomal CTLA-4 loss is more liability than therapeutic necessity [83,98]. Third, context-selective targeting—through acidic extracellular pH, early-endosomal dissociation, or Fc optimization—may matter more than simply intensifying systemic blockade [82,98,99,100,101]. Masked or protease-activated platforms are conceptually aligned with this direction, but the strongest full-text evidence still comes from pH-sensitive and Fc-enhanced approaches rather than from mature primary datasets for masked constructs [99,103]. Preliminary clinical safety data for the acid pH-sensitive antibody ONC-392 have been reported in a conference abstract; however, peer-reviewed clinical efficacy data are not yet available to confirm these observations [102]. So far, the main lesson is that safer anti-CTLA-4 therapy will probably come from using CTLA-4 biology more precisely, rather than from simply increasing the intensity of blockade.

### 5.5. What Therapy Reveals About CTLA-4 Biology in Humans

The therapies discussed in Section 5.1, Section 5.2, Section 5.3 and Section 5.4 also help clarify how CTLA-4 functions in humans. Taken together, they show that no single mechanism is sufficient to explain either efficacy or toxicity. This naturally shifts the discussion toward more precise and biomarker-informed modulation of the pathway.

## 6. Toward Precision Modulation of the CTLA-4 Pathway

### 6.1. Biomarkers of Disease Activity and Treatment Response

Several immunophenotypic markers reflect pathway dysfunction in CTLA-4 insufficiency and LRBA deficiency, although none has been formally validated for routine clinical decision-making. Across available cohorts, recurrent abnormalities include expansion of CD21low B cells, reduction of switched memory B cells, and naïve-to-memory T-cell skewing, whereas increased circulating T follicular helper (cTfh) cells with reciprocal reduction of T follicular regulatory (Tfr) cells have emerged as particularly informative markers in LRBA deficiency [57,96,97]. CD21low B cells were elevated in all tested CTLA-4-insufficient patients in the largest cohort study to date [57], whereas a striking cTfh expansion together with Tfr depletion was documented in LRBA deficiency [96]. These markers also appear treatment responsive: abatacept reduces cTfh frequency, CD21low B cells, and activated cytokine-producing CD4+ T cells, broadly paralleling clinical improvement [96,97]. Residual LRBA protein expression may represent an additional simple prognostic marker, because patients with detectable residual expression were alive and had lower disease burden in the largest LRBA cohort [60].

Integrative multi-omic profiling has extended the biomarker landscape beyond conventional flow cytometry. Patients show compensatory upregulation of alternative inhibitory checkpoints, including *LAG3*, *TIGIT*, *HAVCR2*, *ADORA2A*, and *VSIR*, a signature that normalizes predominantly in the lymphoid compartment after abatacept, whereas myeloid-associated dysregulation remains only partially corrected [97]. Plasma proteomics identifies CHI3L1, CXCL13, and CSF1 as shared biomarkers across CTLA-4 insufficiency and LRBA deficiency, while REG3A and TFF3 are enriched in patients with gastrointestinal involvement [97]. *BCL2L1* (BCL-xL), consistent with enhanced survival signaling downstream of CD28, has also emerged as a candidate biomarker for treatment monitoring [97].

For risk stratification, the immune deficiency and dysregulation activity (IDDA) score is currently the best-available composite metric. Originally developed in LRBA deficiency, it was subsequently applied to CTLA-4 insufficiency [60,62]. In LRBA deficiency, a pre-HSCT IDDA below 15 was associated with 100% survival (n = 3), and transplantation within three years of symptom onset significantly improved outcome [60]. In CTLA-4 insufficiency, a pre-HSCT IDDA at or below the cohort-derived cutoff of 23 was associated with improved overall and disease-free survival [62]. Lung involvement emerged as the strongest organ-specific negative predictor in LRBA deficiency, with all deaths in the largest cohort occurring in patients with pulmonary disease [60]. Finally, longer pre-treatment disease duration and broader organ burden predict poorer abatacept response, further supporting early pathway-targeted intervention [54]. No validated molecular biomarker currently predicts individual treatment response. Prospective multicenter studies will be needed to validate these candidate biomarkers before clinical implementation.

### 6.2. Why Do Some Patients Respond While Others Do Not?

The main factors likely influencing response in CTLA-4 pathway disorders are timing of treatment, baseline disease burden, and how reversible the affected tissue or process still is. In the largest therapeutic cohort to date, abatacept achieved sustained complete control in 46 of 58 treated patients (79.3%), whereas patients with partial or no response had a significantly longer disease duration before treatment than complete responders [54]. The same principle emerges in transplantation cohorts: in LRBA deficiency, hematopoietic stem cell transplantation (HSCT) within three years of symptom onset was associated with significantly better survival, and in CTLA-4 insufficiency lower pre-HSCT IDDA scores predicted superior overall and disease-free survival [60,62]. These data indicate that delayed intervention narrows the therapeutic window as cumulative immune-mediated and structural damage accrues.

Response heterogeneity is also manifestation specific. Abatacept most consistently benefits active immune dysregulation—including lymphoproliferation, cytopenias, enteropathy, and inflammatory organ infiltration—whereas established structural damage is less reversible [61]. This clinical pattern is mirrored by systems-level data: abatacept normalizes lymphoid-associated transcriptional programs much more effectively than myeloid-associated ones, suggesting that some disease compartments remain only partially CTLA-4 dependent once pathology is established [97]. Similarly, cTfh expansion and Tfr deficiency, which reflect active adaptive immune dysregulation, decline with CTLA-4-Ig-based therapy and appear more readily reversible than fixed end-organ injury [96].

Finally, response also depends on the nature of the underlying defect. In LRBA deficiency, residual LRBA protein is associated with lower disease burden and better survival, whereas lung involvement is a particularly strong negative prognostic marker, being present in all deceased patients in the largest cohort [60]. Multi-omic data further show that LRBA deficiency and CTLA-4 insufficiency differ in the depth of dysregulation and in the degree of normalization achieved with abatacept [97]. By contrast, DEF6 deficiency remains too rare for meaningful response-stratification analysis. Patients respond best when treatment is introduced early, before irreversible tissue damage develops, and when pathology remains predominantly driven by active, CTLA-4-dependent immune dysregulation rather than by downstream structural injury.

### 6.3. Modifier Genetics and Incomplete Penetrance

One of the most striking features of CTLA-4 pathway disease is that a clear molecular defect does not translate into a uniform clinical phenotype. In CTLA-4 haploinsufficiency, penetrance is incomplete across major cohorts, ranging from approximately 60.6% in family-based systematic aggregation [58] to 67.6% (90/133) in the largest cohort [57]. Here again, the higher estimate refers to affected individuals among mutation carriers in a clinically referred cohort, whereas the lower estimate derives from family-based aggregation; these values therefore arise from different ascertainment strategies and should not be read as directly interchangeable. Even within the same family, carriers of the same mutation may differ markedly in age at onset, organ involvement, and overall severity [55,56]. This variability suggests that the CTLA-4 defect is necessary, but not sufficient on its own to explain the full clinical phenotype.

The strongest evidence for this comes from asymptomatic carriers. Schubert et al. showed that unaffected mutation carriers can display the same reduction in CTLA-4 expression as clinically affected relatives, and Schwab et al. similarly found reduced CTLA-4 expression and defective trans-endocytosis across tested carriers [56,57]. At the same time, no robust genotype–phenotype correlation has been established: mutation class does not reliably predict penetrance, age at onset, or clinical pattern [57,58]. Thus, the clinical phenotype must be shaped by factors beyond the primary CTLA-4 lesion itself.

The factors underlying this variability remain poorly understood. Modifier genes, epigenetic influences, and environmental or infectious triggers have all been proposed, but no published study has identified HLA or any other specific genetic modifier of penetrance in CTLA-4 pathway disorders [56,57,58,59]. In LRBA deficiency, residual protein expression appears to function as a form of pathway reserve, being associated with lower disease burden and better survival [60]. Whether analogous variation in residual pathway output contributes to penetrance in CTLA-4 haploinsufficiency or to irAE susceptibility during checkpoint blockade remains plausible but unproven [59].

### 6.4. Unresolved Mechanistic Questions

Despite major advances, several mechanistic questions in CTLA-4 biology remain unresolved in humans. One of the most important unresolved questions concerns trans-endocytosis. CTLA-4-mediated removal of CD80 and CD86 has been demonstrated in cell lines, in cocultures with human dendritic cells, and in murine models in vivo [23,25]. Patients with CTLA-4 haploinsufficiency show impaired trans-endocytosis ex vivo [56], providing indirect human evidence but not direct demonstration of this process in human tissues in vivo. However, direct visualization or quantification of trans-endocytosis occurring in human tissues in vivo has not been achieved. A related gap concerns soluble CTLA-4 (sCTLA-4). Although an alternatively spliced soluble isoform is detectable and functionally active in experimental systems, its physiological importance in humans, and whether it contributes meaningfully to immune regulation or instead reflects broader CTLA-4 transcriptional activity, remain uncertain [38]. The difficulty of measuring CTLA-4 recycling at the tissue level compounds both problems: because CTLA-4 resides predominantly in intracellular vesicles and cycles rapidly to and from the surface, simple surface staining underestimates the functionally relevant pool [25], and no validated method currently quantifies this dynamic process directly in human tissues.

In cancer immunotherapy, the extent and importance of intratumoral regulatory T-cell depletion in humans also remain unresolved. Mouse studies clearly show that Fc-dependent Treg depletion and CTLA-4 antagonism can be experimentally separated and that both contribute to maximal efficacy [79,80]. In humans, however, the evidence remains indirect, relying mainly on FcγR genotype-response associations, translational models, and immune profiling rather than on direct quantitative proof of antibody-dependent intratumoral Treg depletion in vivo [79,81]. The same is true for the interplay among ligand blockade, Fc biology, and antibody-induced CTLA-4 degradation or recycling: these mechanisms can be partially disentangled experimentally in mice, but not yet cleanly in patients [80,83,98].

These uncertainties have direct therapeutic consequences. Abatacept controls disease in many patients with CTLA-4 pathway disorders, whereas HSCT can be curative but carries substantial risk; however, there is still no mechanistic framework that reliably explains when pathway replacement is sufficient and when immune dysregulation has become too extensive or too tissue-destructive for abatacept alone [54,60,62,97]. CTLA-4 is now better viewed as a trafficking- and context-dependent pathway rather than as a simple inhibitory receptor, yet several of its most important mechanistic links remain unresolved in humans.

### 6.5. Future Directions in CTLA-4-Targeted Therapy

The future of CTLA-4-targeted therapy lies in moving beyond broad pathway blockade or replacement toward more precise modulation of the pathway that preserves the beneficial aspects of CTLA-4 biology. In oncology, this includes pH-sensitive antibodies that allow endosomal dissociation and CTLA-4 recycling, as well as Fc-engineered molecules designed to enhance intratumoral Treg depletion while limiting systemic toxicity [79,82,83,98,101]. In transplantation, selective CD28 blockade offers a complementary strategy by interrupting costimulation without simultaneously disabling endogenous CTLA-4 function [77]. In monogenic CTLA-4 pathway disorders, the challenge is different but conceptually parallel: therapy must be matched to disease architecture, distinguishing reversible immune dysregulation—often amenable to abatacept—from irreversible tissue injury that may require HSCT [54,62]. Across these settings, future progress will likely depend on biomarker-guided treatment choices and on measuring pathway function more directly, rather than relying only on genotype or phenotype. The goal, therefore, is not simply stronger CTLA-4 intervention, but more precise control of the pathway in each clinical setting.

## 7. Conclusions

CTLA-4 is better viewed as a regulatory pathway than as a static inhibitory receptor. Across Treg cells, Tfr cells, conventional CD4+ T cells, and CD8+ T cells, its function depends on when and where it reaches the cell surface, how it handles CD80/CD86, and how strongly cell-extrinsic control contributes in each setting. This broader view helps explain why disruption of the same pathway can present as monogenic immune dysregulation, polygenic autoimmunity, transplant rejection, antitumor immunity, or immune-related adverse events.

Across the relevant literature, surface availability may be the most useful unifying concept. Gene dosage, ligand binding, endosomal sorting, LRBA- and DEF6-dependent recycling, and antibody-induced lysosomal loss all converge on a practical question: how much CTLA-4 is available, at the right time and place, to control CD80/CD86-dependent costimulation. This helps explain why CTLA-4 haploinsufficiency, LRBA deficiency, and DEF6 deficiency overlap clinically, why CTLA-4-Ig in transplantation does not fully preserve physiological coinhibition, and why anti-CTLA-4 antibodies in cancer can produce efficacy at the cost of systemic toxicity.

Clinically, this means that stronger intervention is not necessarily better. The goal is to match each intervention to the biology of the setting: functional pathway replacement when endogenous CTLA-4 is insufficient, selective costimulation blockade when physiological coinhibition should be preserved, and more precise anti-CTLA-4 strategies in oncology that separate intratumoral benefit from peripheral toxicity. More broadly, the CTLA-4 story highlights that immune regulation depends not only on which receptors are present, but also on how they are trafficked and deployed.

## Figures and Tables

**Table 1:** Monogenic disorders of the CTLA-4 pathway: comparative overview.

Feature	CTLA-4 Haploinsufficiency (CHAI)	LRBA Deficiency	DEF6 Deficiency
Gene	*CTLA4*	*LRBA*	*DEF6*
Inheritance	Autosomal dominant	Autosomal recessive	Autosomal recessive
Molecular mechanism	Reduced CTLA-4 dosage or function (haploinsufficiency with nonsense-mediated decay, or variants impairing ligand binding or structural stability)	Defective LRBA-dependent recycling with accelerated lysosomal degradation of CTLA-4	Defective RAB11-dependent CTLA-4 trafficking to the cell surface
Functional bottleneck	↓ CTLA-4 surface expression and impaired trans-endocytosis	↓ CTLA-4 surface expression (post-translational loss)	↓ RAB11+CTLA-4+ vesicles → ↓ CTLA-4 surface availability
Penetrance	Incomplete: 60.6% in family-based systematic aggregation [58] and 67.6% (90/133) in the largest cohort [57]	High but not absolute; rare asymptomatic individuals have been reported [58,59,60]	Unknown (7 patients aggregated across two very small series) [21,22]
Reported patients	Hundreds of cases reported across overlapping cohorts and reviews [55,56,57,58,61]	Hundreds of cases reported across overlapping cohorts and reviews [19,54,58,60]	7 [21,22]
Predominant phenotype	CVID-like: hypogammaglobulinemia, lymphoproliferation, autoimmune cytopenia, GLILD, enteropathy, and neurologic involvement [57]	Similar spectrum, usually more severe; chronic diarrhea, failure to thrive, and type 1 diabetes [58,59]	Severe early-onset enteropathy, systemic autoimmunity (ANCA, anticardiolipin), hepatosplenomegaly [21]
Genotype–phenotype correlation	No robust correlation established [56,57,58]	No robust correlation established; residual LRBA protein is associated with a milder course [60]	Not assessable; too few published cases [21,22]
Abatacept response	79.3% sustained CR in the combined LRBA/CTLA-4 cohort (46/58) [54]; organ-specific responses in GLILD, gastrointestinal, and CNS disease [61]	79.3% sustained CR in the combined LRBA/CTLA-4 cohort [54]; effective for lymphoproliferation, cytopenia, and enteropathy [19,54]	1/1 sustained remission reported [21]
HSCT overall survival	76.7% at 3 years (40 patients) [62]	70.8% (17/24) [60]	Not reported [21]
Key references	[55,56,57,58,61]	[19,54,58,60]	[21,22]

Abbreviations: CR, complete response; GLILD, granulomatous-lymphocytic interstitial lung disease; HSCT, hematopoietic stem cell transplantation. Penetrance estimates are cohort-dependent and not directly interchangeable across different study designs. Cumulative patient totals are described qualitatively because published cohorts overlap substantially.

**Table 2:** Therapeutic modalities in CTLA-4 pathway disorders: evidence summary.

Therapy	Mechanism of Action	Principal Evidence	Response/Outcome	Key Limitations
Abatacept (CTLA-4-Ig)	Exogenous ligand sequestration: binds CD80/CD86 and functionally substitutes for a key output of endogenous CTLA-4	[54] (n = 58); [61] (n = 29); [19,97]	79.3% sustained CR [54]; GLILD 7/10, gastrointestinal disease 9/9, and cytopenia 24/29 improved [61]; lung disease improvement maintained for up to 7 years [19]	Does not restore the intracellular CTLA-4 reservoir or reverse established tissue damage [62,97]; CD28 blockade may reduce Treg pools [72,97]; nonresponders often had longer disease duration and greater organ burden [54]
Sirolimus ± abatacept	mTOR inhibition ± pathway replacement	[54,60]	May help control severe or refractory disease; lower IDDA scores were reported in observational cohorts [54,60]	Uncontrolled data; no head-to-head comparison
Conventional immunosuppressants	Various (corticosteroids, MMF, rituximab, and others)	[54] (n = 61)	27.9% CR; 72.1% partial or no response [54]	Inferior to abatacept; escalation to abatacept or HSCT was often required
HSCT	Potentially definitive correction of the genetic defect	[62] (CTLA-4); [60] (LRBA); [54] (LRBA)	3-year OS 76.7% in CTLA-4 insufficiency [62] and 70.8% in LRBA deficiency [60]; lower pre-HSCT IDDA was associated with better outcome [60,62]; 70.6% of LRBA survivors were treatment-free [60]	Significant early TRM [60]; pre-existing tissue damage may be irreversible [62]; higher disease burden and lung involvement predict poorer outcome [60,62]; mixed chimerism and graft failure remain risks [62]

Abbreviations: CR, complete response; HSCT, hematopoietic stem cell transplantation; IDDA, immune deficiency and dysregulation activity; MMF, mycophenolate mofetil; OS, overall survival; TRM, transplant-related mortality. In LRBA deficiency, a pre-HSCT IDDA below 15 was associated with 100% survival (n = 3) [60]. In CTLA-4 insufficiency, a pre-HSCT IDDA at or below 23 was associated with improved outcome [62]. In the LRBA cohort, survival after HSCT appeared better in the post-2015 era, although this difference was not statistically significant [60].

**Table 3:** Anti-CTLA-4 antibody strategies in oncology: mechanisms and design principles.

Strategy	Representative Agent(s)	Primary Mechanism of Action	Effect on CTLA-4 Recycling	irAE Profile	Antitumor Efficacy (Preclinical)	Key Refs
Conventional 1st-generation (IgG1)	Ipilimumab	Steric blockade plus FcγR-dependent intratumoral Treg depletion	Drives lysosomal degradation	++++	++	[7,78,79]
Conventional 1st-generation (IgG2)	Tremelimumab	Steric blockade with weaker Fc-dependent intratumoral Treg depletion	Likely degradation-prone (TremeIgG1 model)	+++	++	[79,83]
pH-sensitive (endosomal dissociation)	HL12/HL32; Ipi25; Treme156/157	Early-endosomal dissociation preserves recycling while retaining Fc-mediated Treg depletion	Preserved	+	+++	[83,98]
Acidic pH-selective (TME targeting)	pH Ipi; ONC-392/gotistobart	Acidic-TME-selective or recycling-preserving CTLA-4 targeting with reduced peripheral engagement	Not directly tested for pH Ipi; recycling-preserving design claimed for ONC-392	+	++	[99,102]
Fc-enhanced	Botensilimab	Enhanced FcγR engagement with greater Treg depletion and myeloid activation	Likely degradation-prone (inferred)	++	+++	[82]
Fc-optimized + TA-HEV induction	Fc-optimized anti-CTLA-4	Fc-dependent TA-HEV induction with improved T-cell recruitment and PD-1 sensitization	Not specifically studied	?	+++	[101]
Non-antagonistic depleter	b1s1e2-Fc	Fc-dependent Treg depletion without CTLA-4 antagonism	Not applicable	Not tested	+	[80]
Dual-function (antagonism + depletion)	9d9-mIgG2c	Combined CTLA-4 antagonism and intratumoral Treg depletion	Likely degradation-prone (inferred)	Not separately tested	+++	[80]

Abbreviations: irAE, immune-related adverse event; PD-1, programmed cell death protein 1; TA-HEV, tumor-associated high endothelial venule; TME, tumor microenvironment. The qualitative irAE scale (+ to ++++) is schematic and summarizes relative toxicity reported in the cited preclinical and clinical literature. A key design insight from Zhang et al. is that dissociation at pH 6.0 in early endosomes, rather than generic acidity or weaker affinity alone, is the critical determinant of recycling preservation and therapeutic index [83,98]. For tremelimumab, lysosomal degradation was demonstrated experimentally using a chimeric IgG1 variant (TremeIgG1) rather than native IgG2 [83]. Recycling/degradation assignments for botensilimab and 9d9 are inferred from shared binding logic and were not directly tested in the efficacy studies [80,82]. Most next-generation strategies listed here remain supported predominantly by preclinical data; the ONC-392/gotistobart citation [102] refers to early conference-abstract clinical data.

## Data Availability

No new data were created or analyzed in this study. Data sharing is not applicable to this article.

## Abbreviations

ABACHAI, abatacept in CTLA-4 insufficiency or LRBA deficiency trial; AIHA, autoimmune hemolytic anemia; ANCA, antineutrophil cytoplasmic antibody; APC, antigen-presenting cell; CHAI, CTLA-4 haploinsufficiency; CNI, calcineurin inhibitor; CNS, central nervous system; CR, complete response; CTLA-4, cytotoxic T-lymphocyte-associated protein 4; cTfh, circulating T follicular helper cell; CVID, common variable immunodeficiency; DEF6, differentially expressed in FDCP 6 homolog; DSA, donor-specific antibody; eGFR, estimated glomerular filtration rate; EE, early endosome; FcγR, Fc gamma receptor; FOXP3, forkhead box P3; GC, germinal center; GI, gastrointestinal; GLILD, granulomatous-lymphocytic interstitial lung disease; HSCT, hematopoietic stem cell transplantation; ICI, immune checkpoint inhibitor; IDDA, immune deficiency and dysregulation activity; irAE, immune-related adverse event; ITP, immune thrombocytopenia; LRBA, lipopolysaccharide-responsive beige-like anchor protein; mGFR, measured glomerular filtration rate; mTOR, mechanistic target of rapamycin; OS, overall survival; PBMC, peripheral blood mononuclear cell; PD-1, programmed cell death protein 1; PD-L1, programmed death-ligand 1; sCTLA-4, soluble CTLA-4; TA-HEV, tumor-associated high endothelial venule; TEMRA, terminally differentiated effector memory RA-positive T cell; Tfh, T follicular helper cell; Tfr, T follicular regulatory cell; TME, tumor microenvironment; TRM, transplant-related mortality; Treg, regulatory T cell.
